# Hand MRI and the Greulich-Pyle atlas in skeletal age estimation in adolescents

**DOI:** 10.1007/s00256-017-2867-3

**Published:** 2018-01-25

**Authors:** Azadeh Hojreh, Jutta Gamper, Maria T. Schmook, Michael Weber, Daniela Prayer, Christian J. Herold, Iris-Melanie Noebauer-Huhmann

**Affiliations:** 1 Department of Biological Imaging and Image-guided Therapy, Medical University of Vienna, Waehringer Guertel 18-20, 1090 Vienna, Austria; 2 Centre for Medical Statistics, Informatics and Intelligent Systems, Medical University of Vienna, Spitalgasse 23, 1090 Vienna, Austria

**Keywords:** Skeletal age, Hand MRI, Hand X-ray, Greulich-Pyle atlas, Bone age

## Abstract

**Objective:**

To evaluate the feasibility of hand MRI in age assessment in adolescents using the Greulich-Pyle (GP) atlas criteria.

**Materials and methods:**

Two radiologists, who were blinded to the study subjects’ chronologic ages, semi-objectively evaluated 1.5-T MRIs of the left hands of ten patients (13.5 ± 2.6 years) who had left-hand radiographs and 50 healthy volunteers (15 ± 2 years).

**Results:**

A coronal T1-weighted, volumetric, interpolated, breath-hold examination with water excitation (T1 VIBE-3D-WE) achieved the best image quality. The correlation between estimated patients’ ages on radiographs and MRI was high. The average estimated age difference between the MRIs and radiographs was −0.05 years for reader 1 and −0.175 years for reader 2. The interclass coefficients (ICCs) showed high interobserver agreement (radiographs: ICC = 0.95, MRI: ICC = 0.97). The ICC, calculated separately for the male and female volunteers’ estimated ages by MRI, also showed a high agreement between the two readers (male: ICC = 0.97, female: ICC = 0.95). Reader 1 estimated 94% of volunteers within 2 standard deviations (SD) and 62% within 1 SD. The results for reader 2 were 92% and 54%, respectively. Thirty-nine percent of girls and 27% of boys were estimated to be older using 1 SD.

**Conclusion:**

MRI of the left hand is a feasible alternative to hand radiographs for skeletal age estimation in adolescents using the GP criteria with 2 SD. Using 1 SD, the age of healthy volunteers tended to be estimated as higher than the chronologic age. Future studies should evaluate the results in a larger number of participants.

## Introduction

Since Heinrich von Ranke introduced the use of the hand X-ray to evaluate pediatric growth in 1896, this method has become an important tool in the assessment of the normal and pathologic development of children [1]. Skeletal maturity assessment is clinically essential for pediatric orthopedics in preoperative planning [1, 2], for the diagnosis and treatment of pediatric growth development failure due to congenital or iatrogenic endocrinologic disorders or after chemotherapy or radiation therapy of oncologic patients [3–7]. Repeated annual follow-up hand X-rays (0.0005 mSv effective dose per radiograph) [8], however, result in a not negligible cumulative radiation dose. This method has also been used for forensic age estimation in living individuals [9, 10]. Magnetic resonance imaging (MRI) of the left hand has been preliminarily tested for the assessment of bone maturation [11–16]. Only one pilot study correlated the age estimated by MRI with a radiograph of the left hand [16].

The aim of our study was to evaluate the feasibility of MRI of the left hand to replace the standard radiograph of the left hand in age estimation based on the Greulich-Pyle (GP) hand atlas criteria as the reference standard. The first part of the study aimed to analyze various MR sequences to select the optimal sequence for age estimation and to assess the reliability of MRI of the left hand for skeletal age estimation compared with a standard radiograph of the left hand of patients; the second part aimed to compare the age estimated by MRI with the chronologic age of volunteers.

## Methods and materials

### Patients and healthy volunteers

The local ethics committee approved this prospective study. Written informed consent was obtained from all patients and volunteers or from their parents if they were minors.

Inclusion criteria were informed consent, lack of either MRI contraindications or need of sedation, and furthermore, for volunteers, no past medical history of any chronic diseases. The exclusion criterion was noncompliance during the examination.

In the first part of the study, ten patients (eight males and two females; mean age, 13.5 years; range, 11–18 years) with endocrinologic diseases, in whom a standard radiograph of the left hand had been performed, also underwent an MRI of the left hand within a week of the radiograph. All of the patients were native Europeans (at least two generations of ancestors born in Europe).

In the second part of the study, 50 healthy volunteers (17 males and 33 females; mean age, 15 years; range, 12–19.8 years) underwent an MRI of the left hand. All healthy volunteers were middle-class and born in Europe. Forty-six healthy volunteers were native Europeans (at least two generations of ancestors born in Europe), and the parents of the remaining four volunteers were from Iran, Argentina, Mali, and the Philippines.

### Imaging technique

All radiographs of the left hand in the first part of the study were performed in the dorsovolar projection on an X-ray unit (Polydoros IT Opti 150/12/50 C®, Siemens Healthineers, Siemens Healthcare GmbH, Germany) using 18 × 24-cm Fuji storage phosphor plates and a Fuji Dry Laser machine (Fuji DryPix 7000®, Fujifilm Holdings Corp., Tokyo, Japan).

All MRI examinations were performed on a short 1.5-T closed-bore (bore size 60-cm) scanner (Magnetom Avanto®, Siemens Healthineers, Siemens Healthcare GmbH, Germany), using a head-neck coil combination and in the prone position with the left arm outstretched (superman position). To reduce motion artifacts, a small sandbag was placed on the left hand.

In the first part of the study, three sequences were applied:

1. Coronal T1-weighted turbo-inversion recovery magnitude (T1 TIRM):
  - Matrix: 176 × 384; voxel size: 0.5 × 0.5 × 3.0 mm; field of view (FOV): 200 mm; slice thickness (SL): 3 mm; gap: 0.3 mm; repetition time (TR): 4110 ms; echo time (TE): 47 ms; inversion time: 150 ms; flip angle: 150°; acquisition time: 3 min and 31 sec
2. Coronal T1-weighted volumetric interpolated breath-hold examination with water excitation (T1 VIBE-3D-WE):
  - Matrix: 384 × 512; voxel size: 0.4 × 0.4 × 1.5 mm; FOV: 230 mm; SL: 1.5; gap: 0.3 mm; TR: 14.6 ms; TE: 6.07 ms; flip angle: 15°; acquisition time: 2 min and 26 sec
3. Coronal T1-weighted spin echo (T1 SE):
  - Matrix: 384 × 512; voxel size: 0.4 × 0.4 × 2.0 mm; FOV: 200 mm; SL: 2 mm; gap: 0.2 mm; TR: 523 ms; TE: 23 ms; flip angle: 90°; acquisition time: 5 min and 5 sec

In the second part of the study, only the coronal T1 VIBE-3D-WE sequence was applied, with the same parameters as described above.

The MRI software version “Syngo MR B13 4VB13A” (Siemens Healthineers, Siemens Healthcare GmbH, Germany) was used in the first part of the study and “Syngo MR B17” (Siemens Healthineers, Siemens Healthcare GmbH, Germany) in the second part.

### Evaluation

Two radiologists with 9 (consultant pediatric radiologist, reader 1) and 20 years (senior consultant musculoskeletal radiologist) of experience in musculoskeletal radiology evaluated the three sequences and determined the image quality and usefulness for the assessment of bone maturation parameters using a 10-point scale.

The decision criteria were:

1. Ossification recognition, defined as the delineation of ossified and cartilaginous parts versus joint spaces
2. Diagnostic usefulness, defined as the contrast between the ossified and cartilaginous matrix
3. Overall subjective image quality
4. Motion and other artifacts

The image quality was rated as 1–2 for non-diagnostic, 3–4 for poor, 5–6 for acceptable, 7–8 for good, and 9–10 for excellent quality. For artifacts, the 10-step scale defined 1–2 as non-diagnostic, 3–4 as severe, 5–6 as moderate, 7–8 as mild artifacts, and 9–10 as an absence of artifacts.

Mean values were calculated for all evaluated parameters and the sequence considered the best was used for age estimation in the first and second parts of the study.

In addition, two consultant radiologists with 9 (consultant pediatric radiologist, reader 1) and 8 years (consultant musculoskeletal radiologist, reader 2) of experience in skeletal age estimation performed semiquantitative subjective age estimation on all images, blinded to the chronologic age of the volunteers and patients, based on the parameters of the GP atlas.

All study images were assessed on a picture-archiving and communication system (PACS) station (IMPAX ES, DS 3000©, Agfa Healthcare, Mortsel, Belgium).

In the first part of the study, the readers reviewed the radiographs of the patients’ left hands, and, after a 1-month time interval, the readers reviewed the MRIs of the left hands of these patients using the most useful MRI sequence and then independently determined an estimated age for each patient and each examination.

The evaluation criteria for age estimation were the ossification stages of the epiphysis of the radius and ulna, carpal bones, metacarpal bones, thumb extensor and flexor sesamoid, and phalanges.

In the second part of the study, the two readers reviewed the MRIs of the left hands of healthy volunteers using the same evaluation criteria as in the first part of the study and independently estimated the skeletal age of each volunteer. In addition, males and females were analyzed separately.

### Statistical analysis

Bland-Altman plots [17] for both readers were drawn for the ten patients to determine agreement between radiographs and MRIs. Estimates for the mean difference between radiographs and MRIs and for the 95% limits of agreement (defined as the mean difference ± 1.96 SD of the difference) were calculated. For all estimates, 95% confidence intervals (CI) were calculated. Intraclass correlation coefficients (ICC) were calculated for radiographs and for MRIs to determine agreement between readers 1 and 2.

For the age estimation of the healthy volunteers, Bland-Altman analysis for both readers was performed for males and females separately. The standard deviation in the Greulich-Pyle atlas is provided only up until 17 years (boys) and 15 years of age (girls). For volunteers outside that range, the last available standard deviation was assumed. ICCs were also calculated for male and female volunteers separately to determine agreement between the readers in both groups.

## Results

All examinations were well tolerated and completed with no compliance issues.

**Fig. 1:**
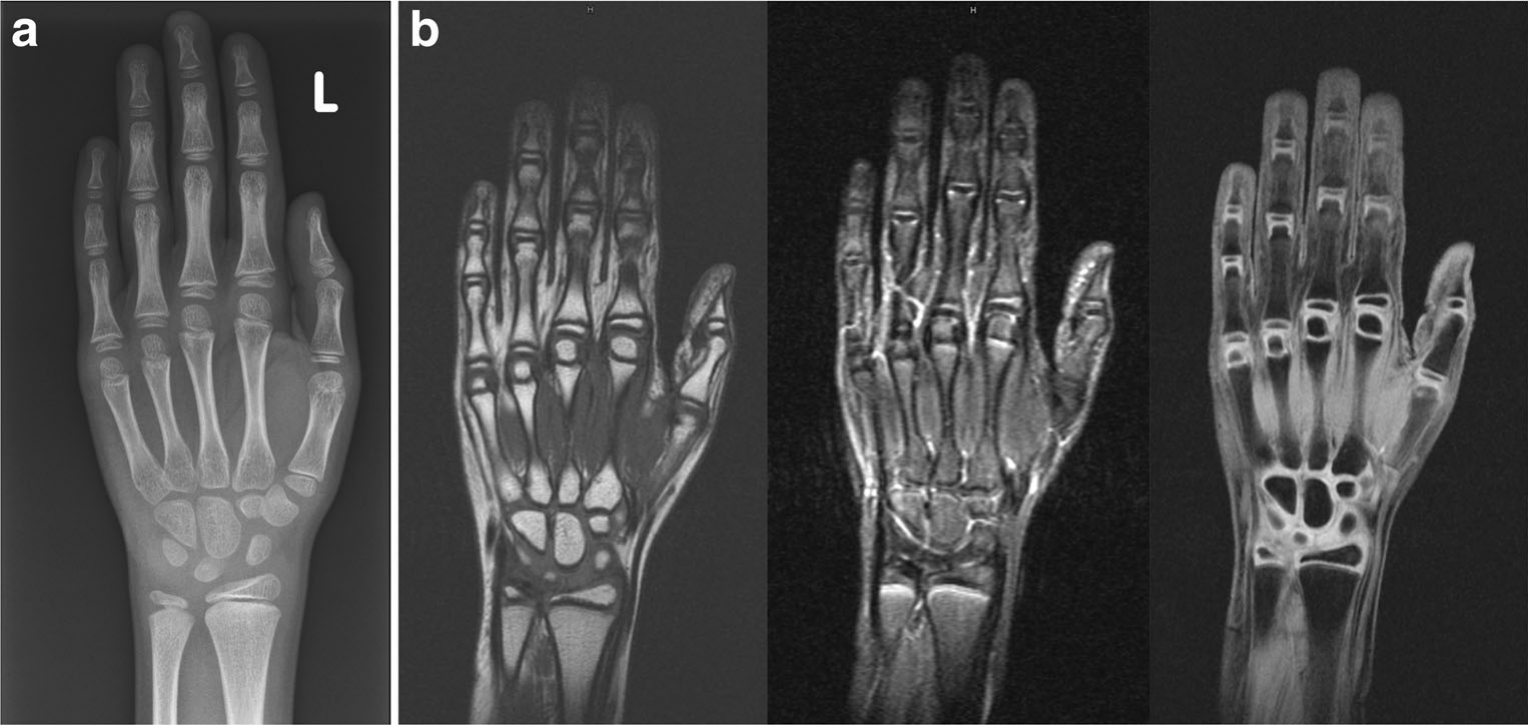
(a) Radiograph of the left hand and (b) coronal T1 SE, T1 TIRM, and T1 VIBE-3D-WE (from left to right) of an 11-year-old male with growth failure. The image quality of T1 VIBE-3D-WE was evaluated as better than the other sequences

Comparison of the three different sequences by subjective evaluation revealed the best values for the coronal T1 VIBE-3D-WE, including visibility of the anatomic structures (Table 1 and Fig. 1a and b). As T1 VIBE-3D-WE was also the shortest sequence (acquisition time 2 min and 26 sec), it was chosen for age estimation for the first and second parts of the study.

**Table 1:** Results of the evaluation of the diagnostic usefulness of the three coronal hand MRI sequences of ten patients in the first part of the study by reader 1 and the senior radiologist

Readers	Sequences	Ossification recognition		Diagnostic usefulness	Subjective image quality	Artifacts	
		Delineation of ossified parts	Delineation of cartilaginous parts vs. joint spaces	Contrast between ossified and cartilaginous matrix		Motion	Other
Reader 1	T1 TIRM	3.4	2.4	2.4	2.6	9.5	10.0
	T1 VIBE^1^	9.8	9.5	10.0	10.0	10.0	10.0
	T1 SE	8.6	6.6	7.9	8.7	10.0	10.0
Senior radiologist	T1 TIRM	4.2	1.0	1.7	4.1	9.5	10.0
	T1 VIBE^1^	9.9	10.0	10.0	8.8	10.0	10.0
	T1 SE	8.9	5.9	7.8	8.5	10.0	10.0
Mean	T1 TIRM	3.8	1.7	2.1	3.4	9.5	10.0
	T1 VIBE^1^	9.8	9.7	10.0	9.4	10.0	10.0
	T1 SE	8.8	6.2	7.9	8.6	10.0	10.0

^1^T1 VIBE-3D-WE

**Fig. 2:**
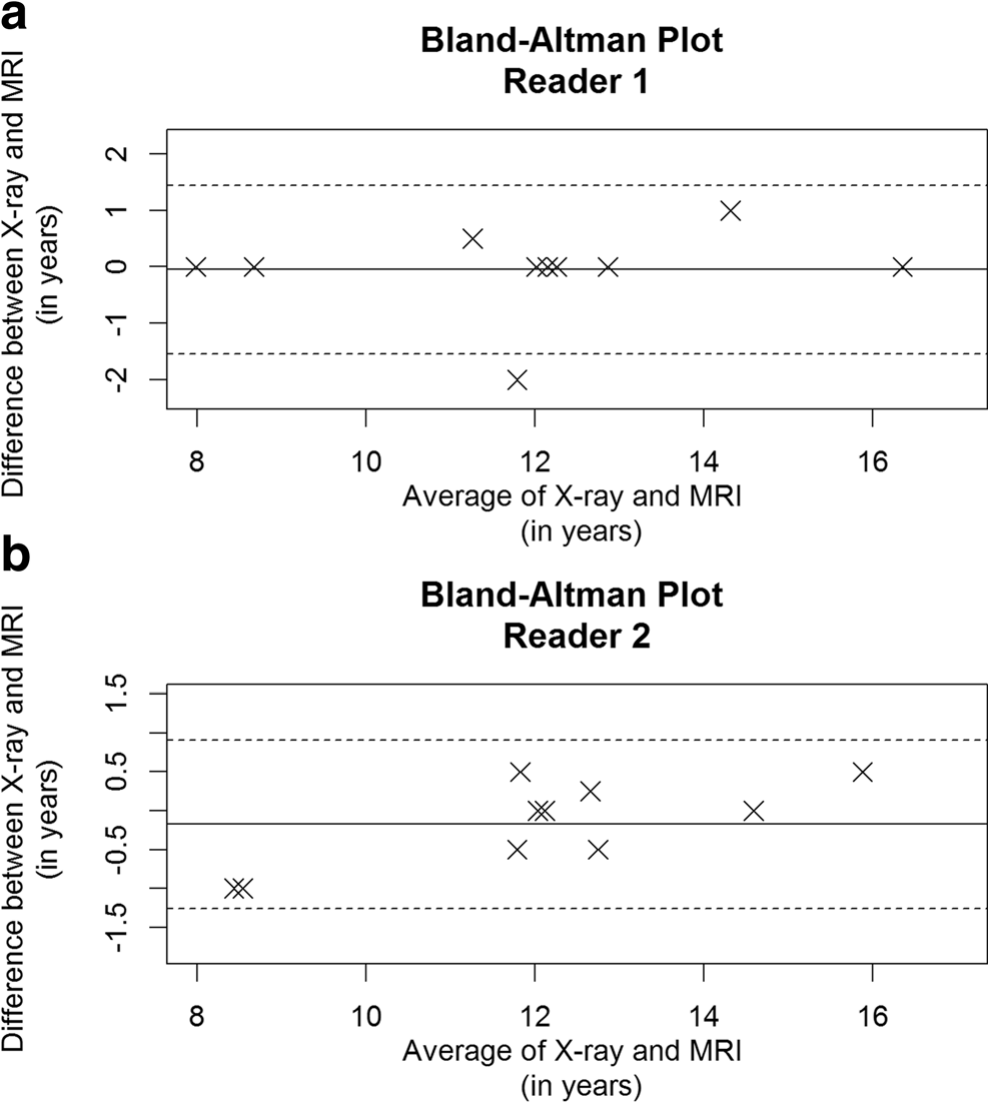
**a** and **b** The Bland-Altman plots for both readers in the first part of the study. The plots show the mean of the estimated ages from the two methods (radiographs and MRIs) on the x-axis, given in years, and the difference between the estimated ages on the y-axis, also given in years. The dotted lines give 95% limits of agreement for estimated ages (average difference ± 1.96 SD of the difference)

In the first part of the study, the comparison of age estimation by radiographs and MRIs revealed a high correlation between these methods (Table 2). The Bland-Altman plots for both readers are provided in Fig. 2a and b.

**Table 2:** Estimated ages for ten patients in the first part of the study for radiographs and MRIs of the left hand, using the GP^1^ atlas (sorted by sex)

Patient no.	Sex	Reader 1 X-ray	Reader 1 MR	Reader 2 X-ray	Reader 2 MR
1	m	9	9	8	9
3	m	15	14	14.5	14.5
4	m	8	8	8	9
5	m	12.5	12.5	12.5	13
6	m	11.5	11	12	12
7	m	12.5	12.5	12.75	12.5
9	m	12.5	12.5	12.5	12
10	m	16	16	16	15.5
2	f	12	12	12	12
8	f	11	13	12	11.5

^1^Greulich-Pyle atlas

The Bland-Altman plot for reader 1 shows that the estimated ages with MRIs and radiographs matched in seven children. The average age difference between MRIs and radiographs was −0.05 years (95% CI: -0.60; 0.40), and the limits of agreement ranged from −1.54 years (95% CI: -2.08; −1) to 1.44 years (95% CI: 0.90; 1.98). Only one patient was estimated to be 2 years older by the MRI compared with the radiograph. For reader 2, the differences between estimated ages by MRI and radiograph were ± 1 year. The average age difference was −0.175 years (95% CI: -0.57; 0.22), and, compared with reader 1, the range of the limits of agreement was narrower, from −1.26 years (95% CI: -1.65; −0.87) to 0.91 years (95% CI: 0.52; 1.30).

It was expected that 95% of the differences would lie between these limits. As the range of the limits of agreement is not clinically important, the two methods can be used interchangeably [17]. ICC showed a high agreement for the two readers: 0.95 for radiographs and 0.97 for MRIs.

In the second part of the study, a comparison of the estimated age by hand MRI with the true chronologic age of the healthy male and female volunteers was made, and the results are presented in Table 3.

**Table 3:** Estimated ages in the second part of the study for MRIs of the left hand of volunteers using the GP^1^ atlas (sorted by sex and chronologic or true age of the volunteers)

Volunteer no.	Sex	True age	Estimated age using hand MR		Volunteer no.	Sex	True age	Estimated age using hand MR	
			Reader 1	Reader 2				Reader 1	Reader 2
41	f	19.8	18^#^	18^#^	2	m	18.5	19	19
47	f	19.8	18^#^	18^#^	40	m	18	19	19
1	f	18.7	18^#^	17	11	m	17.1	17	17
33	f	17.9	18	18	25	m	16.5	19	19
8	f	17.6	17	17	28	m	16.5	19	19
46	f	17.5	18	18	35	m	16.3	18	17
32*	f	17.3	18	18	48	m	16	17	17
50	f	17.1	18	18	4	m	15.8	16	17
24	f	16.6	18	18	16	m	15.3	15	15
34	f	16.2	18	18	21	m	15	17	17
10	f	16.0	18	18	5	m	13.6	13	14
23	f	15.4	14	13.5	27	m	13.6	13	13
12	f	15.1	16	15	9	m	13.3	13	13
14*	f	14.9	16	16	36	m	13	13	13
49*	f	14.7	15	16	39	m	12.9	12.5	11.5
13	f	14.4	15	15	22	m	12.8	12	11
30	f	14.3	13.5	15	19	m	12.3	13	14
7	f	14	13	13					
43	f	14	17	17					
42	f	14	16	15					
17	f	13.8	15	15					
15	f	13.7	16	16					
18	f	13.5	13.5	13.5					
44	f	13.4	13	13					
45	f	13.2	13	13.5					
3	f	13.2	15	15					
6*	f	13.1	15	15					
37	f	13.1	12	13					
38	f	13	10	11.5					
31	f	12.8	15	13					
20	f	12.6	14	14					
29	f	12.5	13	12					
26	f	12.0	11	10					

^1^Greulich-Pyle atlas

^*^Volunteers with a non-European background

^#^Last GP image of adult skeleton (18 years or older)

**Fig. 3:**
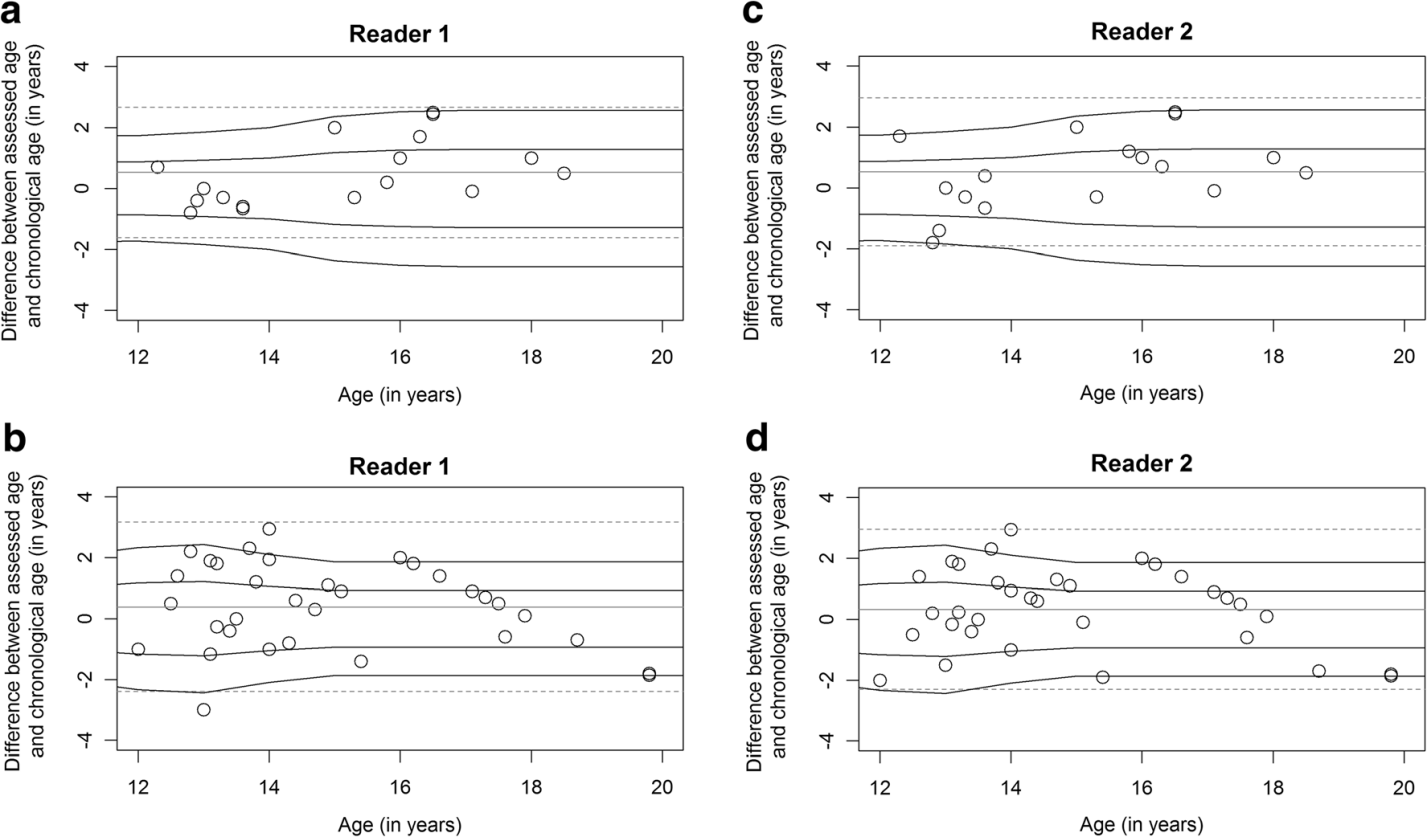
**a–d** The Bland-Altman plots for both readers in the second part of the study for (a) males and (b) females for reader 1 and for (c) males and (d) females for reader 2. The chronologic age of the volunteers is shown on the x-axis and the differences between the assessed age and chronologic age on the y-axis, given in years. The dotted lines correspond to 95% limits of agreement. The continuous lines present the 1st and 2nd SD related to the volunteers’ ages

The Bland-Altman plots for both readers are given in Fig. 3a–d (for males: Fig. 3a and c and for females: Fig. 3b and d).

Of the 15 male volunteers in the age group where the standard deviation (SD) was available in the GP atlas, 11 (73%) and 8 (53%) were estimated correctly within 1 SD, and 15 (100%) and 14 (93%) were estimated correctly within 2 SD by reader 1 and reader 2, respectively. The two male volunteers with a chronologic age of 18 years or older were estimated at 19 years of ages by both readers. Of the 22 female volunteers in the age group where the SD was available in the GP atlas, 12 (55%) were estimated correctly within 1 SD, and 20 (91%) were estimated correctly within 2 SD by both readers. The three female volunteers with a chronologic age of 18 years or greater were all estimated at 18 years of age by reader 1, and only one of the three was estimated at 17 years of age by reader 2.

Using 1 SD, the ages of nine females (41%) were estimated to be older than their chronologic ages, and the age of only one female (5%) was estimated to be younger than her chronologic age by reader 1. Reader 2 estimated eight females (36%) to be older than their chronologic age und two females (9%) younger than their chronologic age. Four males (27%) were estimated to be older than their chronologic age by both readers. No males were assessed younger than their chronologic ages by either reader.

We did not find any consistent bias for adjusting the estimated ages by MRI (Fig. 3a–d).

The ICC, calculated separately for males and females, also showed high agreement between the two readers for both groups (males: ICC = 0.97; females: ICC = 0.95).

**Fig. 4:**
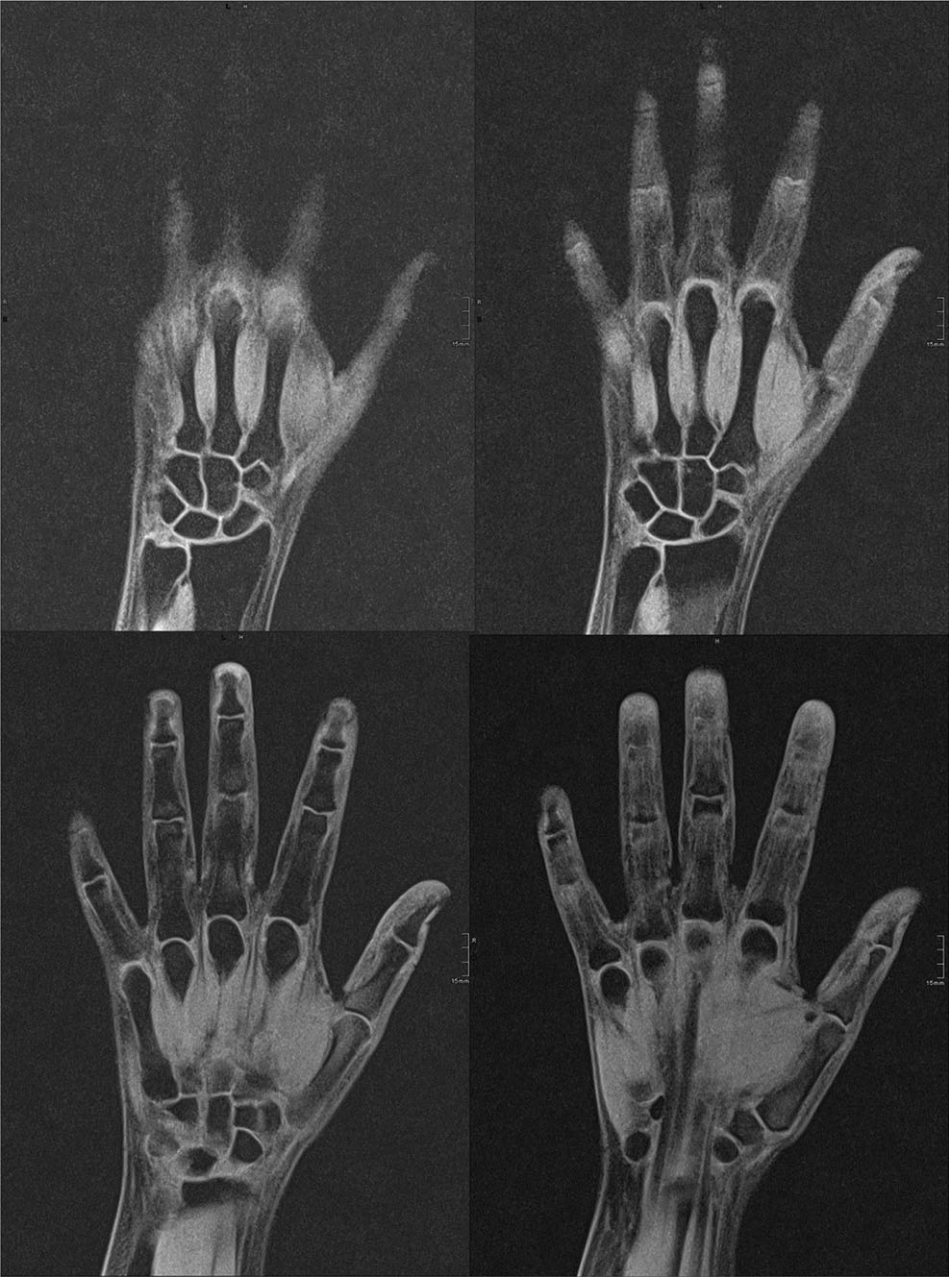
Four slices of a coronal T1 VIBE-3D-WE hand MRI of a 16-year-old healthy female. Estimated age was 18 years (reader 1) and 18 years (reader 2)

**Fig. 5:**
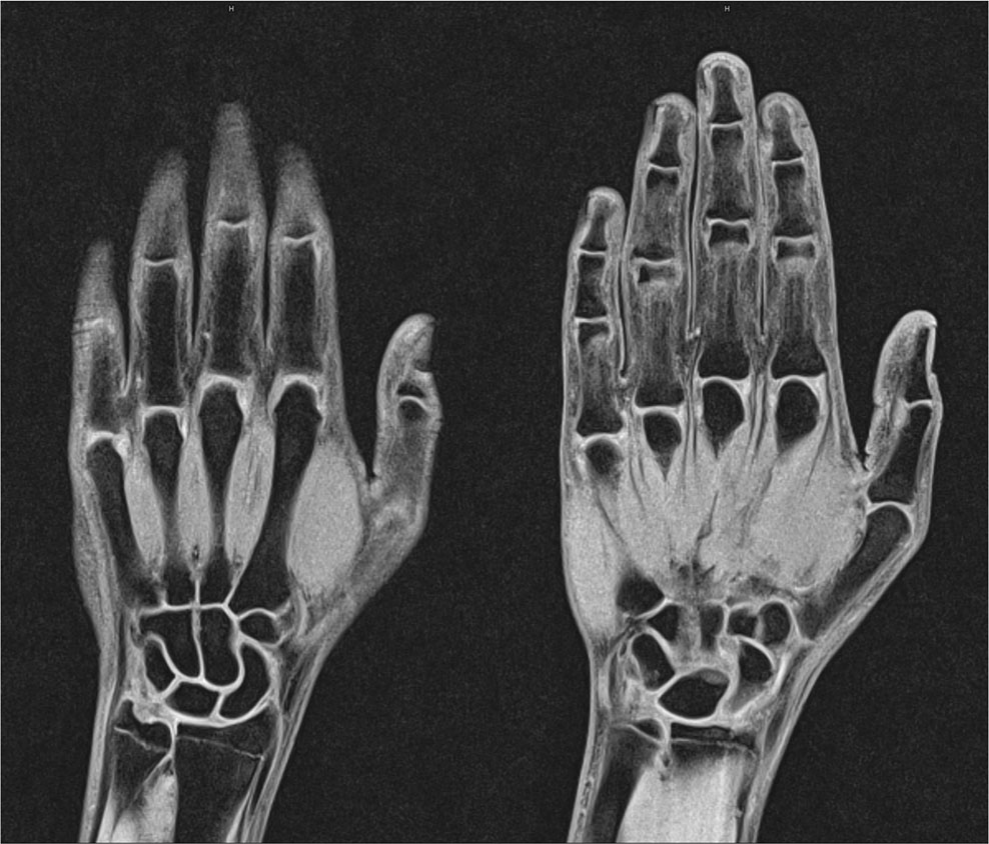
Two slices of a coronal T1 VIBE-3D-WE hand MRI of a 16-year-old healthy male. Estimated age was 17 years (reader 1) and 17 years (reader 2)

The MR images of two healthy volunteers are presented in Figs. 4 and 5.

## Discussion

In our study, we estimated the bone age by MRI and a radiograph of the left hand in accordance with GP criteria. In skeletal age estimation, a radiograph of the left hand is chosen rather than of the right hand, because the number of right-handed persons in most populations is much larger than that of left-handed individuals; consequently, the left hand is less likely to be injured than the hand that is used more frequently [18]. Dreizen et al. compared radiographs of left and right hands and stated that the divergences of the skeletal maturations of the two hands are negligible in the evaluation of skeletal status [19]. Roche found that the left hand was more advanced than the right hand [20], while Baer and Djrkatz found no such effect [21]. An automated age estimation of the metacarpals found no significant difference between the two sides [22].

Subjective systematic evaluation of three optimized high-resolution sequences (T1 TIRM, T1 VIBE-3D-WE, T1 SE) by two consultant radiologists with different years of experience revealed the best values for the coronal T1 VIBE-3D-WE, including visibility of the anatomic structures. It was also the shortest sequence. Short examination times are beneficial to avoid motion artifacts and maintain the compliance of children. Semiquantitative bone age estimation through the assessment of all anatomic landmarks was possible in all 60 subjects of our study with high interobserver agreement at 1.5 T using a single high-resolution T1 VIBE-3D-WE sequence and a head-neck coil combination.

Compared with the fast, low-angle shot three-dimensional fat-suppressed (FLASH 3D–FS) sequence, fat-suppressed 3D VIBE achieved images with a higher cartilage signal-to-noise ratio (SNR), higher contrast-to-noise ratio (CNR) between the cartilage and the surrounding tissues, and reduced pulsation artifacts in a much shorter acquisition time [23].

Tomei et al. used a low-field open magnet (0.2 T) with a hand and wrist coil and applied a coronal single T1 SE sequence with a scan time of 1 min 39 s [11]. Terada et al. also used a low-field (0.3-T) open compact magnet system and applied a 3D gradient echo (GRE) sequence, with a scan time of 2 min 44 s [14]. The choice of the magnet, however, depends on the availability within the environment. We used the 1.5-T short closed-bore magnet, which is used for diagnostic imaging in daily clinical practice, for both adults and children. In general, high-field closed-bore (1.5-T) MR scanners provide higher spatial and contrast resolution than do open low-field (0.2-T) scanners [24] and have revealed considerably higher image quality with less image noise as well as a higher SNR with a shorter scanning time [25].

We also found high agreement between the estimated bone ages using the coronal T1 VIBE-3D-WE of the left hand and the dorsovolar hand X-ray, based on the criteria of the GP atlas, for both consultant radiologists.

This is in contrast to George et al., who compared the fusion grades of the left wrist distal radial growth plate between MRIs and radiographs [15]. In their study, the bone age of 15–19-year-old male football players was estimated as higher with radiographs compared with MRIs [15]. However, the study focused on the radial growth plate of the left wrist and a single criterion only and used a coronal T1-weighted sequence [15]. Urschler et al. compared the estimated ages in 18 subjects using radiographs and MRIs of the left hand and stated that, in subjects between 14 and 18 years of age, the estimated ages by MRI were slightly lower than on a radiograph [16]. A possible explanation for these differences could be that the growth plate is better visible on cross-sectional imaging, such as MRI, than in projection radiographs. In the first part of our study, the average difference between estimated ages by MRI and a radiograph was not significant.

In the second part of the study, we also found high interobserver agreement for age estimation in healthy boys and girls using the coronal T1 VIBE-3D-WE of the left hand based on the GP criteria. This is in accordance with Tomei et al., who used a single T1 SE sequence [11]. We used the GP criteria, as this standard has been proven superior to Tanner and Whitehouse in terms of the time required to determine skeletal age and thus has been recommended for routine clinical practice [1]. The GP criteria also provide high interobserver agreement for age estimation for hand X-rays and hand MRIs compared with those of Tanner and Whitehouse [16].

We also observed that more than 90% of estimated bone ages for healthy volunteers (males and females) were within 2 SD (94% for reader 1 and 92% for reader 2). This corresponds to the GP atlas results using hand X-rays [18]. However, using 1 SD, our results (62% for reader 1 and 54% for reader 2) differed from the GP atlas results for hand X-rays, in which approximately two-thirds of the estimated ages were within 1 SD [18]. Although our volunteers were from the socioeconomic middle class with a background of good medical care, similar to those assessed by the GP atlas, which comprised X-rays of healthy white children, we also found that in the age group with an available SD, healthy volunteers also tended to be estimated as older than their chronologic age using 1 SD. As an overestimation of skeletal maturation would have implications for legal and medical procedures, such as in the diagnosis and treatment of growth failure, further studies with a larger number of study subjects should be performed.

The difference between our results and the GP results using 1 SD is likely attributable to skeletal maturation changes since 1950, reflecting the different standard deviations compared with the GP atlas, but may also be influenced by the small number of cases in our study. To our knowledge, there are no published data that address skeletal maturation changes from 1950 to the present at this time. Future studies should evaluate the results in a larger number of participants.

### Study limitations

Our results were based on a small number of cases and focused also on children older than 10 years, who were cooperative during the examination, without any sedation. No comparison of ethnicities was performed. The gender distribution was asymmetric in both parts of the study. However, because the evaluation of boys and girls was performed separately, the results of the study should not be biased. Multicenter studies are necessary to confirm the study statements in a larger number of participants.

## Conclusion

In conclusion, MRI of the left hand, using a single coronal sequence, T1 VIBE-3D-WE, in a routine scanner is a radiation-free alternative method feasible for skeletal age estimation of adolescents using the GP-based criteria. The age of healthy adolescents could be correctly estimated by expert readers within 2 SD by hand MRI and the Greulich-Pyle atlas, with high agreement. However, when using only 1 SD, bone ages tend to be estimated as older than the chronologic ages. Future studies should evaluate the results in a larger number of participants.
